# Cancer and Lhermitte-Duclos disease are common in Cowden syndrome patients

**DOI:** 10.1186/1897-4287-8-6

**Published:** 2010-06-17

**Authors:** Douglas L Riegert-Johnson, Ferga C Gleeson, Maegan Roberts, Krysta Tholen, Lauren Youngborg, Melvin Bullock, Lisa A Boardman

**Affiliations:** 1 Mayo Clinic, Jacksonville, Florida, USA; 2 Mayo Clinic, Rochester, Minnesota, USA; 3 University of Florida, Jacksonville, Florida, USA

## Abstract

**Background:**

Cancer risk and Lhermitte-Duclos disease (LDD) risk estimates for Cowden syndrome (CS) are broad and based on a small number of patients. Risk estimates are vital to the development of diagnostic criteria, genetic counseling, and cancer surveillance. To further elaborate and estimate the risks associated with CS, a large cohort of patients was evaluated.

**Methods:**

CS patients were identified from the medical literature and the Mayo Clinic's records. All patients met accepted diagnostic criteria for CS.

**Results:**

A total of 211 CS patients (age 44 ± 16 years, 64% female, 46% *PTEN *mutation) were included (published literature 90% and Mayo Clinic series 10%). The cumulative lifetime (age 70 years) risks were 89% for any cancer diagnosis (95% confidence interval (CI) = 80%,95%), breast cancer [female] 81% (CI = 66%,90%), LDD 32% (CI = 19%,49%), thyroid cancer 21% (CI = 14%,29%), endometrial cancer 19% (CI = 10%,32%), and renal cancer 15% (CI = 6%,32%). A previously unreported increased lifetime risk for colorectal cancer was identified (16%, CI = 8%,24%). Male CS patients had fewer cancers diagnosed than female patients and often had cancers not classically associated with CS. Seven percent of breast and thyroid cancers occurred in patients who were younger than the recommended age to commence radiographic cancer screening. There was a trend for patients with a family history of CS and *PTEN *mutations to have a lower cancer risk than those without.

**Conclusions:**

This study confirms CS patients are at increased risk for cancer and quantitative data is provided to guide clinical care. Based on a different tumor spectrum, separate male and female clinical CS diagnostic criteria may be indicated.

## Background

Cowden syndrome (CS) is an autosomal dominant disorder characterized by benign hamartomas and an increased risk of breast, thyroid, and other cancers (Table 1) [1]. CS is one of several syndromes associated with germline mutations in the *PTEN *gene, including Bannayan-Riley-Ruvalcaba syndrome, Proteus or Proteus-like syndrome, adult Lhermitte-Duclos disease (LDD), and autism-like disorders associated with macrocephaly [2-5].

**Table 1:** Diagnostic criteria for Cowden syndrome in individuals without a family history

Pathognomonic criteria	Lhermitte-Duclos disease (dysplastic cerebellar gangliocytoma) Mucocutaneous features: Six or more facial papules, ≥3 must be trichilemmomas, or Cutaneous facial papules and oral mucosal papillomatosis, or Oral mucosal papillomatosis and acral keratoses, or Six or more palmo-plantar keratoses
Major criteria	Breast cancer Thyroid cancer (especially follicular) Endometrial cancer Macrocephaly (≥ 97% percentile)
Minor criteria	Other structural thyroid lesions (for example, multinodular goiter) Mental retardation (IQ ≤ 75) Gastrointestinal hamartomas Fibrocystic breast disease Lipomas Fibromas Genitourinary tumors (examples include, uterine fibroids and renal cell carcinoma) Genitourinary structural malformations

Diagnostic criteria for Cowden syndrome is met in individuals without a family history if there are any of the following: 1) Adult Lhermitte-Duclos disease or the required mucocutaneous features; 2) Two major criteria 3) One major and three minor criteria; 4) Four minor criteria. Adapted from Eng [15]. See Eng for complete criteria.

Previously reported CS associated cancer risk profiles have varied and are usually illustrated with wide variations. The commonly quoted risks are 25-50% for breast cancer, 5-10% for endometrial cancer, and 3-10% for thyroid cancer [6]. These estimates are based on a collection of case series, each often comprising a small number of patients. In addition to cancer, CS patients are at risk for LDD. LDD is the eponym for a dysplastic cerebellar gangliocytoma, which is pathognomonic for CS. The incidence of LDD in CS is unclear. LDD is usually considered to be a hamartoma and not a cancer, but may still have serious consequences and therefore was included in this study.

As there is a limited evidence base for CS cancer and LDD risks, counseling patients regarding their risks and appropriate surveillance is challenging. This study aims to clarify these issues by reporting cumulative cancer and LDD diagnoses in a large group of CS patients.

## Methods

The patient cohort was derived from both the published medical literature and the records of the Mayo Clinic (Rochester, Minnesota). The Mayo Clinic Institutional Review Board (IRB) approved this study. Patients from the medical literature were identified by searching http://www.pubmed.com using the terms "Cowden's syndrome" and "Cowden syndrome." Further reports were identified by reviewing the references listed in the initial publications retrieved. Mayo Clinic CS patients were identified through a search of medical records from January 1, 1996 to June 30, 2008 for any patients with the keyword "Cowden" in their records.

Inclusion criteria required all patients to meet accepted diagnostic criteria for CS (Table 1). Patient data was entered into the study database by one author. The database was audited for accuracy by the other authors by reviewing the source information (literature or medical record) for each patient. Importantly, all cases were reviewed to determine if they had been published more than once (duplicate publication). Where the same patient had been published more than once, only the most recent report was included. In some medical literature cases, the age of cancer diagnosis was unclear (for example, "patient had thyroid cancer in their 20 s"). In these cases, the authors made a best estimate. Statistical analysis was performed with JMP 8.0 (SAS Institute, Cary, North Carolina).

## Results

A total of 211 patients were included in the final version of the study database, 90% from the literature (n = 190) and 10% from the Mayo Clinic (n = 21). Cumulative lifetime (age 70 years) risk estimates were made with 210 patients; one literature patient had to be excluded as age data was not available. The literature patients were identified from review of 99 publications. Thirty one additional publications were identified in the literature search for review but were unavailable from the Mayo Medical Library or via inter-library loan. To date, information pertaining to individuals from the Mayo Clinic series had not been published. Patient demographics revealed that the majority were female (64%, n = 136) with a mean age at most recent follow up of 44 ± 16 years.

**Figure 1:**
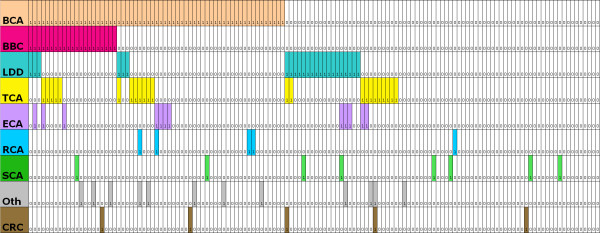
**A clinical array of the cancer and Lhermitte-Duclos disease (LDD) diagnoses in the 136 female Cowden syndrome patients included in the study**. Each column of rectangles represents an individual patient. A diagnosis is indicated by a shaded box containing a "1". Rows represent cancer or LDD diagnosis: BCA (pink) - breast cancer (n = 61 cases); BBC (magenta) - bilateral breast cancer (n = 21 cases); LDD (teal) - Lhermitte-Duclos disease (n = 24 cases); TCA (yellow) - thyroid cancer (n = 23 cases); ECA (violet) - endometrial cancer (n = 12 cases); RCA (blue) - renal cancer (n = 5 cases); SCA (green) - skin cancer (n = 8 cases); Oth (grey) - other cancers (n = 12 cases); CRC (brown) - colorectal cancer (n = 5 cases). Patients were sorted into groups and subgroups by cancer diagnosis (hierarchical sorting). This aids identification of the clustering of one cancer diagnosis with another. Patients were first sorted into those with and without breast cancer. Those with breast cancer were then sorted into those with and without bilateral breast cancer. Each of the subgroups (breast cancer, bilateral breast cancer, and no breast cancer) were then sorted into those with and without LDD and then into those with and without thyroid cancer. Figure formatting taken from Faghri and others [14].

**Figure 2:**
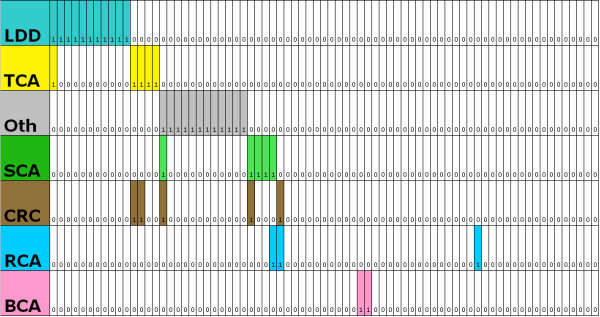
**A clinical array of the cancer and Lhermitte-Duclos disease (LDD) diagnoses in the 75 male Cowden syndrome patients included in the study**. See the caption of Figure 1 for an explanation of figure color coding and formatting. The diagnoses were LDD n = 11 cases, thyroid cancer n = 5 cases, other cancers n = 12 cases, skin cancer n = 5 cases, colorectal cancer n = 5 cases, renal cancer n = 3 cases, and breast cancer n = 2 cases. Patients were sorted into groups and subgroups by cancer diagnosis (hierarchical sorting). Patients were first sorted into those with and without LDD. Then those with LDD were sorted into those with and without thyroid cancer. Next the subgroups were sorted into those with and without other cancers, followed by those with and without skin cancer.

**Figure 3:**
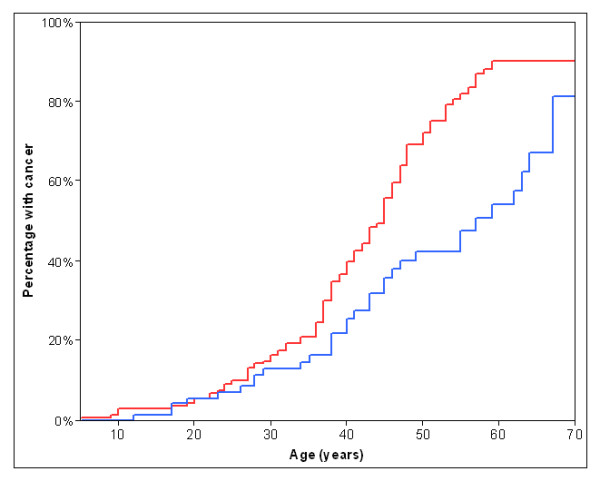
**Cumulative risk of any cancer diagnosis in female (red) and male (blue) patients with Cowden syndrome from birth to age 70 (Kaplan-Meier)**.

**Figure 4:**
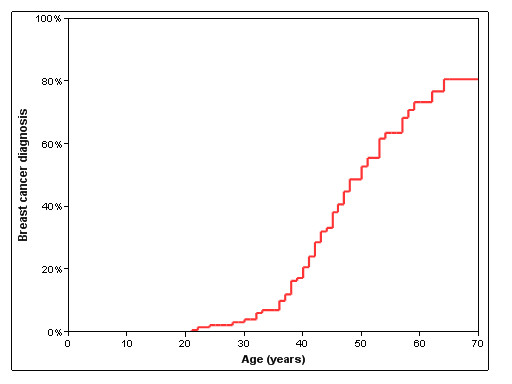
**Cumulative risk of breast cancer diagnosis in female patients with Cowden syndrome (CS) from birth to age 70 (Kaplan-Meier)**. Sixty-one of 136 female CS patients and 2 of 75 male CS patients were diagnosed with breast cancer.

Most patients had been diagnosed with cancer or LDD (62%, n = 130), and a quarter (24%, n = 50) had been diagnosed with either two cancers or a cancer with a LDD diagnosis. Clinical arrays were made to show the cancer and LDD diagnoses in the study patients (Figure 1 - **females**; Figure 2 - **males**). The cumulative lifetime cancer risks are highlighted in Table 2 and Figure 3. The most commonly reported cancer was breast cancer in females (Figure 4). Of female CS patients diagnosed with breast cancer, 34% had bilateral disease (n = 21). In the 15 cases of bilateral breast cancer where there was adequate data to determine metachronous or synchronous status, 8 were synchronous and 7 were metachronous. Several rare cancers were reported in one or only a few CS patients including testicular germ cell tumor and trichilemmomal carcinoma [7,8].

**Table 2:** Cumulative cancer risk by age and site in 210 patients with Cowden syndrome (95% confidence intervals)

Site	20 yrs	30 yrs	40 yrs	50 yrs	60 yrs	70 yrs
**All cancers**	9% (6-13)	18% (13-24)	35% (29-43)	63% (46-70)	78% (70-85)	89% (80-95)
**Breast [female]**	0	4% (2-10)	19% (12-27)	53% (42-63)	73% (61-83)	81% (66-90)
**Lhermitte-Duclos**	4% (2-8)	10% (6-15)	13% (9-19)	21% (15-28)	21% (15-28)	32% (19-49)
**Thyroid**	4% (0-5)	5% (10-29)	9% (6-15)	17% (12-25)	21% (14-29)	21% (14-29)
**Endometrium**	1% (0-5)	1% (0-5)	1% (0-5)	9% (5-19)	19% (10-32)	19% (10-32)
**Colon and rectum**	0	0	0	3% (1-10)	13% (7-25)	16% (8-24)
**Kidney**	0	1% (0-4)	1% (0-4)	3% (1-8)	7% (3-16)	15% (6-32)

Risks were estimated using Kaplan-Meier estimation. Table format from Hearle and others [16].

The cumulative CS cancer risks (but not overall cancer risks) found in this study were higher than the ranges reported in the literature (Table 3). The Mayo Clinic and medical literature cases had a similar total percentage of cancer/LDD cases (62% and 61%, respectively). The most striking difference between the Mayo Clinic and literature cases was the diagnosis of LDD. LDD was not observed in any Mayo Clinic CS cases, while it was diagnosed in 15% of the literature cases.

**Table 3:** Cowden syndrome and general population cancer risks

Site	Literature†	This study: Overall	This study: Cumulative* (95% CI)	General population lifetime risk‡
**Breast [female]**	25-50%	45%	81% (66-90)	14%
**Lhermitte-Duclos**	Not reported	15%	32% (19-49)	Very rare
**Thyroid**	3-10%	12%	21% (14-29)	1%
**Endometrium**	5-10%	9%	19% (10-32)	3%
**Colon and rectum**	Not reported	5%	16% (8-24)	6%
**Kidney**	Not reported	4%	19% (10-32)	1%

* Cumulative lifetime (age 70) risks (95% confidence intervals)**. † **From Pilarski [6]. **‡ **General population lifetime cancer risks, except for LDD, are from the National Cancer Institute's Surveillance Epidemiology and End Result (SEER) database (http://seer.cancer.gov/csr/1975_2004/results_merged/topic_lifetime_risk.pdf, accessed August 25^th^, 2009). The risks cited include both invasive cancer and in-situ disease.

Less than half of male CS patients had a cancer or LDD diagnosis compared to the majority of females with CS (males 45% vs. females 70%). Many males with a cancer diagnosis did not have cancers usually associated with CS (i.e. LDD, breast, thyroid, and renal cancer) (Figure 2). These cancers included three cases of lung adenocarcinoma and solitary cases of carcinoid, testicular seminoma, testicular cancer, trichilemmomal carcinoma, prostate adenocarcinoma, hepatocellular carcinoma, melanoma, transitional cell carcinoma of the bladder, anal squamous cell cancer.

The screening commencement age to encompass 100% of cases varied between cancer types (Table 4). The largest difference was between LDD and colorectal cancer; to capture 100% cases of LDD, screening would need to begin at age 9, while the detection of 100% of colorectal cancers would require screening beginning at age 43. The number of additional years of screening needed to improve from 95 to 100% case capture was 3-7 years except for endometrial cancer where 10 years would be needed.

**Table 4:** Ages to initiate screening to capture 95 and 100% of reported cancer cases by site

Site	95% of cases (yrs)	100% of cases (yrs)
**Breast [female]**	28	21
**Lhermitte-Duclos disease**	14	9
**Thyroid**	17	10
**Endometrium**	32	22
**Colon and rectum**	46	43
**Kidney**	33	28

The age that encompasses 100% of cancer cases for a particular site is also the age of the youngest reported case for that site.

A family history or a possible family history of CS was noted in 53% (n = 111). A *PTEN *mutation was identified in 46% of patients overall and in 92% of those that had *PTEN *testing (97 of 105 were mutation positive). The spectrum of *PTEN *mutations reported was nonsense (41%, n = 40), missense (14%, n = 14), splice site and other (12%, n = 12), and mutation found but type not noted (32%, n = 31). In patients who had *PTEN *testing, one of four patients *without *an identifiable mutation had breast cancer compared to 24 of 64 *with *an identifiable mutation being diagnosed with breast cancer (25% vs. 38%, p = 0.5 Fisher's exact test).

There was no obvious association of the diagnosis of one cancer with the diagnosis of another cancer (as an example, patients who were diagnosed with breast cancer were no more likely to be diagnosed with thyroid cancer than patients who did not have breast cancer) (Figures 1 and 2). Family history of CS or a reported *PTEN *mutation were less likely to occur in the cancer group, but this trend was not statistically significant (family history odds ratio (OR) 0.6 95% confidence interval (CI) = 0.4, 1.1; *PTEN *mutation OR 0.7, 95% CI = 0.4, 1.1).

## Discussion

We report that cumulative lifetime CS cancer risks are higher than those previously published. Previously reported risks were overall estimates from small groups of patients. The large cohort of patients studied in this report made it possible to determine cumulative risks with adjustment for the number of patients at risk at a particular age (Kaplan-Meier estimation). The presented findings highlight the importance of determining cumulative lifetime risks in hereditary cancer syndromes.

Ascertainment and publication biases both may have artificially elevated the risks reported here. Evidence of publication bias includes that the proportion of patients with LDD was much higher in the medical literature than the unpublished Mayo Clinic series. As cases of LDD are rare, they are more likely to be published as case reports. Ascertainment and publication bias can be corrected for by excluding probands from the analysis. This approach would have left only 23 patients; a number too small for meaningful analysis.

The National Cancer Comprehensive Network (NCCN) issues the most widely used cancer surveillance guidelines for CS (Table 5) [9]. The findings of this study confirm the current NCCN guidelines as the initial starting point for prescribing an individual CS cancer surveillance program. The age at which to begin cancer screening is a key question in hereditary cancer syndrome surveillance (the interval for testing is assumed to be one year and the test is usually an imaging modality or an endoscopy). In the authors' collective experience, patients and care providers initially request screening beginning at the age of the youngest reported case of cancer. The NCCN recommends mammography and breast magnetic resonance imaging (MRI) to commence at 30 - 35 years for most CS patients (those without a family history of breast cancer). For thyroid cancer surveillance, NCCN recommends an initial thyroid ultrasound at age 18 years. Seven percent of CS breast and thyroid cancers occurred prior to the recommended onset of radiographic screening by NCCN. In order to capture 100% of reported breast and thyroid cancer cases with imaging, breast screening would need to commence at age 21 (9 years younger than recommend by NCCN) and thyroid screening at age 10 (8 years younger than recommended by NCCN). Lowering the recommended age of screening to encompass all reported cases, results in screening a group with only a small chance of being diagnosed with cancer. The authors often utilize data from studies such as this one to illustrate this concept for patients. For example, if an annual breast MRI was performed on this study's female cohort from ages 21 (the age of the youngest reported case) to 30 (the recommended NCCN start age), approximately 800 breast MRIs would be performed in a cohort with only four breast cancer diagnoses (0.5%). Also, about 80 of the 800 breast MRIs (10%) would require a recall of the patient for further testing possibly including breast biopsy [10].

**Table 5:** Cancer surveillance guidelines for Cowden syndrome

General	Physical examination beginning at age 18 or 5 years before the diagnosis of breast or thyroid cancer in the family.
Thyroid	Baseline ultrasound thyroid examination at age 18 and consider yearly thereafter.
Skin	Annual dermatologic exam.
Colon	None.
Breast [female]	For those without a family history of breast cancer, self breast examination and clinical breast examination are recommended to commence at ages 18 and 25, respectively. Also, mammography and breast magnetic resonance imaging are recommended to begin at ages 30 to 35. Consider prophylactic mastectomy.
Endometrial [female]	Educate on symptoms and consider enrollment in a clinical trial.

Adapted from the National Comprehensive Cancer Network (NCCN) guidelines for Cowden syndrome [9]. In addition to the NCCN recommendations, the authors recommend colon and rectum cancer screening with colonoscopy to begin at age 45 and every 5 years thereafter.

CS patients often have colorectal polyps, and it has been unclear if these polyps place such patients at increased risk for colorectal cancer (CRC) [6,11]. Current recommendations are that CS patients should have average risk CRC screening with colonoscopy beginning at 50 years of age [9,12]. The elevated CRC risk identified here would support earlier and more intense screening. The authors recommend colonoscopy beginning at age 45 and every 5 years thereafter.

This study did not identify any risk factors for cancer in CS patients. This is consistent with the findings from most other hereditary cancer syndromes. The patients who had a family history consistent with CS had a trend for lower cancer risk than those without a family history. The most likely reason for this is, ascertainment bias; patients with a family history were more likely to be identified by non-malignant CS signs such as skin findings rather than cancer. There was also a trend for lower cancer risk in those with a *PTEN *mutation. This is possibly the result of individuals suspected to have CS but who do not have a cancer diagnosis having *PTEN *testing to confirm the diagnosis.

An association between an identified *PTEN *mutation and breast cancer diagnosis in CS has been reported [13]. Our study does not support this association. The utility of a detailed genotype/phenotype analysis of this study's database is limited and was not performed for several reasons. These include that only half of patients had *PTEN *testing and of those with a mutation identified most had a nonsense mutation limiting the diversity of mutation types.

Cancer and LDD are critical components to recognizing and diagnosing CS and the current CS diagnostic criteria includes LDD, breast, thyroid, renal and endometrial cancers. Most males did not have cancer and many that did had cancers that are not usually associated with CS. Developers of the clinical criteria for CS diagnosis should consider separate criteria for male and female patients with the male criteria having a decreased emphasis on cancer diagnosis and inclusion of cancers not usually associated with CS. Regardless of the diagnostic criteria used, clinicians should maintain a high degree of suspicion for the often subtle signs of CS.

## Competing Interests

The authors declare that they have no competing interests.

## Authors' contributions

DRJ and LB conceived the study. KT, LY, and FG populated the study database. MB prepared Figures 1 and 2. DRJ preformed the statistical analysis and drafted the manuscript. FG, MR and LB provided critical review. All authors read and approved the manuscript.
